# Different Subcomponents of Executive Functioning Predict Different Growth Parameters in Mathematics: Evidence From a 4-Year Longitudinal Study With Chinese Children

**DOI:** 10.3389/fpsyg.2018.01037

**Published:** 2018-06-21

**Authors:** Wei Wei, Liyue Guo, George K. Georgiou, Athanasios Tavouktsoglou, Ciping Deng

**Affiliations:** ^1^College of Education, Shanghai Normal University, Shanghai, China; ^2^Shanghai Key Laboratory of Brain Functional Genomics, Changning-ECNU Mental Health Center, School of Psychology and Cognitive Science, East China Normal University, Shanghai, China; ^3^Department of Educational Psychology, University of Alberta, Edmonton, AB, Canada; ^4^Faculty of Science, Concordia University of Edmonton, Edmonton, AB, Canada

**Keywords:** executive functioning, working memory, arithmetic, mathematics, Chinese, longitudinal

## Abstract

Executive functioning (EF), an umbrella term used to represent cognitive skills engaged in goal-directed behaviors, has been found to be a unique predictor of mathematics performance. However, very few studies have examined how the three core EF subcomponents (inhibition, shifting, and working memory) predict the growth parameters (intercept and slope) in mathematics skills and even fewer studies have been conducted in a non-Western country. Thus, the purpose of this study was to examine how inhibition, shifting, and working memory predict the growth parameters in arithmetic accuracy and fluency in a group of Chinese children (*n* = 179) followed from Grade 2 (mean age = 97.89 months) to Grade 5 (mean age = 133.43 months). In Grade 2, children were assessed on measures of nonverbal IQ, number sense, speed of processing, inhibition, shifting, and working memory. In addition, in Grades 2–5, they were assessed on arithmetic accuracy and fluency. Results of structural equation modeling showed that nonverbal IQ, speed of processing, and number sense predicted the intercept in arithmetic accuracy, while working memory was the only EF subcomponent to predict the slope (rate of growth) in arithmetic accuracy. In turn, number sense, speed of processing, inhibition, and shifting were significant predictors of the intercept in arithmetic fluency. None of the EF subcomponents predicted the slope in arithmetic fluency. Our findings reinforce those of previous studies in North America and Europe showing that EF contributes to mathematics performance over and above other key predictors of mathematics, and suggest that different EF subcomponents may predict different growth parameters in mathematics.

## Introduction

Executive functioning (EF), an umbrella term used to represent cognitive skills engaged in goal-directed behaviors, such as inhibition, mental flexibility, and working memory (e.g., Chan et al., 2008; Best et al., 2009; Diamond, 2013), is important not only for behavioral regulation in classroom that ultimately enhances learning (e.g., Day et al., 2015), but also for the development of specific cognitive skills that further support children’s academic performance (e.g., Fuhs et al., 2016). One of the academic skills that EF appears to make a unique contribution to is mathematics (e.g., Espy et al., 2004; Blair and Razza, 2007; Willoughby et al., 2012; Allan et al., 2014). Despite the acknowledged importance of EF in mathematics performance, far less is known about how EF subcomponents predict the growth parameters (intercept and slope) of mathematics development. Therefore, the purpose of this study was to examine how the three core EF subcomponents (inhibition, shifting, and working memory) predict the growth parameters of two different mathematics skills (arithmetic accuracy and fluency) in a sample of Chinese children followed from Grade 2–5.

Executive functioning has been conceptualized as a multicomponent construct composed of inhibition, shifting, and working memory (e.g., Miyake et al., 2000; Lehto et al., 2003; Wu et al., 2011; Xu et al., 2013; however, see also Testa et al., 2012, for more EF subcomponents). Inhibition, defined as the ability of an individual to override a dominant but inappropriate response, may help children suppress inappropriate strategies while operating on math problems or suppress the prepotent activation of an inappropriate number representation (Bull and Lee, 2014). In turn, shifting, defined as flexibly switching attention between different mindsets, may help individuals switch between different operation rules. Finally, working memory, defined as the ability of an individual to hold information in short-term memory (storage) while processing some other (processing), is needed when solving different mathematics problems [e.g., (3 + 5) ^∗^ 4 = ?] because individuals need to first hold part of the solution in their memory (e.g., the result of 3 + 5) before executing another operation (e.g., multiplying by 4).

Among the three EF subcomponents, working memory is perhaps the most studied in relation to mathematics (see Raghubar et al., 2010, for a review). Two meta-analyses have reported a moderate correlation between working memory and different mathematics skills (*r*s ranged from 0.31 to 0.38 in Friso-van den Bos et al., 2013; the average correlation was 0.35 in Peng et al., 2015). Recent studies have also shown that inhibition and shifting are significant correlates of mathematics performance (e.g., Blair and Razza, 2007; Andersson, 2008; Clark et al., 2010; Willoughby et al., 2012; Gilmore et al., 2015; Cragg et al., 2017; Purpura et al., 2017), although the strength of their relationship appears to be lower compared to that of working memory. In their meta-analyses, Allan et al. (2014) and Friso-van den Bos et al. (2013) reported an average correlation of 0.27 between inhibition and mathematics. Likewise, Yeniad et al. (2013) and Friso-van den Bos et al. (2013) found the correlation between shifting and mathematics to be 0.26 and 0.28, respectively.

Unfortunately, most previous EF studies have focused on the role of individual EF subcomponents and, as a result, we do not know how they predict mathematics skills in the presence of each other. In addition, the few studies that have included all three EF subcomponents have produced mixed findings (e.g., Bull and Scerif, 2001; Andersson, 2008; Agostino et al., 2010; van der Ven et al., 2012; Cantin et al., 2016; Lubin et al., 2016; Cragg et al., 2017; Vandenbroucke et al., 2017). For example, whereas some studies have found working memory to account for unique variance in mathematics skills after controlling for the effects of all other EF subcomponents (e.g., Bull and Scerif, 2001; Agostino et al., 2010; Lubin et al., 2016; Cragg et al., 2017; Vandenbroucke et al., 2017), others failed to find any significant effects (e.g., Espy et al., 2004; Cantin et al., 2016). Similarly, whereas some studies have found that inhibition and shifting make a unique contribution to mathematics skills (e.g., Espy et al., 2004; Andersson, 2008; Cantin et al., 2016; Cragg et al., 2017; Purpura et al., 2017), others did not (e.g., Monette et al., 2011; Rose et al., 2011; Lee et al., 2012; Vandenbroucke et al., 2017).

There might be two reasons for the mixed findings. First, they may reflect differential effects of EF subcomponents on different mathematics skills. Mathematics skills consist of several components themselves including arithmetic accuracy (the accuracy of performing different operations either by using procedural or retrieval strategies) and arithmetic fluency (the speed with which different arithmetic problems are solved). In studies in which math accuracy scores were used, working memory was found to make a unique contribution (e.g., Andersson, 2008; Agostino et al., 2010; Lee et al., 2012; Cragg et al., 2017). In contrast, in studies in which fluency scores were used, working memory did not predict mathematics (e.g., Andersson, 2008; Cantin et al., 2016; Purpura et al., 2017). The opposite pattern appears to be true for inhibition and shifting. Studies have reported a unique contribution of shifting and inhibition to arithmetic fluency (e.g., Andersson, 2008; Clark et al., 2010; Cragg et al., 2017) but not to accuracy (e.g., Agostino et al., 2010; Lee et al., 2012). Cragg and Gilmore (2014) concluded that the contribution of EF subcomponents may differ across different aspects of mathematics skills.

Second, most previous studies examining the contribution of inhibition and shifting to mathematics skills have administered speeded measures of both, without controlling for the effects of speed of processing. As van der Sluis et al. (2007), and more recently Georgiou and Das (2018) have indicated, in this kind of studies unless researchers control for speed of processing we do not know if the effects of EF on mathematics are driven by their executive processing demands or by speed. Most of the EF tasks, especially the inhibition and shifting tasks, are speeded because of ceiling effects in accuracy (e.g., Anderson, 2002; Lee et al., 2012). The results of a meta-analysis by Yeniad et al. (2013) showed that the average correlation between response time measures of shifting and mathematics (*r* = 0.36) was higher than that between accuracy measures of shifting and mathematics (*r* = 0.25). Rose et al. (2011) and Bull and Lee (2014) further argued that the variance in mathematics skills explained by EF may be attributed to speed of processing, because speed of processing, as a domain-general cognitive skill, also contributes to mathematics. Thus, the contribution of EF subcomponents, especially of inhibition and shifting, may decrease when the effects of speed of processing are controlled. In line with this prediction, some studies have found that inhibition was no longer predicting the mathematics skills when the effects of speed of processing were controlled (Rose et al., 2011; Purpura et al., 2017). Fuchs et al. (2006) also showed that working memory did not explain any unique variance in mathematic skills after controlling for the effects of speed of processing and nonverbal IQ. Certainly, these findings need to be replicated.

Beyond the contradictory findings of previous studies that included all three EF subcomponents, previous studies examining the role of EF in mathematics skills suffer from at least three limitations. First, most previous studies have not examined the role of the EF subcomponents in the presence of other key predictors of mathematics such as number sense. Number sense refers to an individual’s “fluidity and flexibility with numbers,” which includes skills such as understanding what numbers mean and how they relate to each other (Gersten and Chard, 1999). The first reason why the effects of number sense should be partialled out is that some EF tasks (e.g., Trail Making) typically use numbers as their stimuli and this may inflate the relations with mathematics (Cragg and Gilmore, 2014). In addition, although some previous studies have shown that earlier EF predicts future number competence (e.g., Kolkman et al., 2013; McClelland et al., 2014; Purpura et al., 2017), little is known about whether EF continues to predict mathematics skills after controlling for number competence such as number sense. Fuhs et al. (2016), for example, found that the effects of early EF on concurrent mathematics performance were fully mediated by number sense, and Simanowski and Krajewski (2017) also found that EF in kindergarten could not predict mathematic skills in Grades 1 and 2 (mean ages were 87 and 99 months, respectively) after controlling for early number competence. Therefore, as Viterbori et al. (2015) have suggested, children’s number sense should be controlled before examining the contribution of EF subcomponents to mathematics skills.

Second, most previous studies examining the relationship between EF and mathematics are cross-sectional (e.g., Agostino et al., 2010; Rose et al., 2011; Cantin et al., 2016). The few longitudinal studies (e.g., Swanson, 2006; McClelland et al., 2014; Simanowski and Krajewski, 2017; Vandenbroucke et al., 2017) have covered only a limited developmental span (most often from Kindergarten to Grades 1 and 2) and have used the EF skills (assessed at an earlier point in time) to predict mathematics skills at a later point in time (often assessed once). To our knowledge, only a handful of longitudinal studies have examined how EF predicts different growth parameters (intercept and slope) in mathematics (see Bull et al., 2008; Geary, 2011; van der Ven et al., 2012; Van de Weijer-Bergsma et al., 2015; Lee and Bull, 2016), and of these studies only two had assessed all three EF subcomponents (Bull et al., 2008; van der Ven et al., 2012). The results of van der Ven et al. (2012) showed that working memory (updating) in Grade 1 (mean age = 77 months) correlated with the intercept in mathematics (a comprehensive mathematics test) during Grades 1 and 2 (mean age = 95 months), while a factor composed of inhibition and shifting in Grade 1 did not correlate with either growth parameter. Similarly, the results of Bull et al. (2008) showed that working memory along with inhibition at kindergarten (mean age = 54 months) predicted the intercept in mathematics during Grade 1 (5–6 years old) and Grade 3 (7–8 years old). Furthermore, Geary (2011) and Lee and Bull (2016) examined the growth parameter of arithmetic accuracy (assessed with numerical operations) during a longer span (more than 3 years), and found working memory also predicted the slope in arithmetic accuracy. Another study, Van de Weijer-Bergsma et al. (2015), found working memory at the beginning of Grade 2 (6–8 years old) correlated with the intercept not the slope in math fluency during Grade 2. Thus, more research is needed on how all three EF subcomponents predict the growth parameters of mathematics development.

Finally, almost all of the studies reviewed above were conducted in North America or Europe and we do not know if their findings generalize to East Asian countries (e.g., China). There are reasons to believe that the role of EF subcomponents may be different in China than in Western countries. The first reason relates to the role of working memory. Because Chinese digits are monosyllabic and have a shorter pronunciation duration they allow individuals to hold a larger number of digits in their short-term memory. If simple calculations can be solved with direct retrieval of facts from long-term memory, then individuals with a larger pool of arithmetic facts in their memory should also have superior performance in calculations. Indeed, a few cross-cultural studies have shown that Chinese outperform North Americans in mental calculation (e.g., Stevenson et al., 1990; Campbell and Xue, 2001; Wang and Lin, 2009; Lonnemann et al., 2016). Imbo and LeFevre (2009) also showed that Chinese university students required fewer working memory resources than Belgian or Canadian university students when solving complex addition problems. If Chinese children solve simple addition and subtraction problems by relying on rote memorization, then the contribution of working memory may not be as strong as it has been reported in previous studies in North America. Some studies have provided evidence in support of this hypothesis (e.g., Geary et al., 1996; Thorell et al., 2013; Cui et al., 2017), but more research is needed.

Second, inhibition may be less important for mathematics skills among Chinese children than among North-American children. Geary et al. (1996) found that more than half of American children in Grade 2 (mean age = 94 months) and Grade 3 (mean age = 104 months) were still using basic strategies in addition, such as counting fingers and verbal counting, while almost all Chinese children in the same grades (with comparable mean ages) were relying on direct retrieval. Relying on strategies such as verbal counting may lead to one of the most common errors in calculation, i.e., a counting-string associate of one of the addends (e.g., 3 + 5 = 6; Siegler and Shrager, 1984). To avoid this error, American children have to actively suppress any irrelevant association when retrieving arithmetic facts from long-term memory. In contrast, Chinese children may not need to inhibit irrelevant associations if they directly retrieve the answers to calculations from their long-term memory. Indeed, Lan et al. (2011) found that inhibition of Chinese preschoolers uniquely predicted counting, but failed to predict calculation, while inhibition of American children uniquely predicted both counting and calculation. Similarly, Peng et al. (2012) found that performance on a color-word Stroop task (one of the most widely used measures of inhibition) failed to differentiate between Chinese fifth-graders with mathematics difficulties and their typically developing peers (the mean age of both groups was 132 months).

To our knowledge, only four studies have examined the contribution of EF to mathematics skills among Chinese children and all of them have focused on the concurrent relationships between some EF subcomponents and mathematics during kindergarten. Three studies with Chinese preschoolers (Zhang, 2016; Chung et al., 2017; Zhang et al., 2017) showed that inhibition and working memory or an EF factor composed of inhibition and working memory uniquely predicted early mathematics skills after controlling for rapid naming, vocabulary, and visual skills. Another study (Lan et al., 2011) found that Chinese preschoolers’ inhibition predicted counting, but failed to predict calculation. Working memory predicted both counting and calculation. Therefore, it remains unclear whether EF subcomponents can predict mathematics skills longitudinally, especially the growth rate of mathematics skills.

### The Present Study

The purpose of this study was to examine how the three core EF subcomponents (inhibition, shifting, and working memory) predict the growth parameters (intercept and slope) of arithmetic accuracy and fluency in a group of Chinese children followed from Grade 2 to 5. Based on the findings of previous studies that examined the predictors of growth parameters in mathematics performance (see Bull et al., 2008; Geary, 2011; van der Ven et al., 2012; Van de Weijer-Bergsma et al., 2015; Lee and Bull, 2016), we expected that:

(1) Working memory would predict both growth parameters of arithmetic accuracy (see Geary, 2011; Lee and Bull, 2016), and the intercept of arithmetic fluency (see Van de Weijer-Bergsma et al., 2015),
(2) Inhibition would predict only the intercept of arithmetic fluency (see Bull et al., 2008) and,
(3) Shifting would not predict any growth parameter in any mathematics skill (see van der Ven et al., 2012).

## Materials and Methods

### Participants

One hundred seventy-nine Grade 2 Chinese children (82 girls and 97 boys; mean age = 97.89 months, *SD* = 3.56) were recruited on a voluntary basis from public schools in Shanghai (China) to participate in the study (T1). The children were reassessed in Grades 3, 4, and 5 (T2, T3, and T4), when they were 109.65 (*SD* = 3.62), 122.99 (*SD* = 3.55) and 133.43 (*SD* = 3.70) months old, respectively. By Grade 5, only 165 children (or 92% of the original sample) remained in the study. The children who withdrew from the study did not differ significantly from the children who remained in the study on any of the measures administered in Grade 2 (all *p*s > 0.10). All children were native speakers of Mandarin and none was experiencing any intellectual, sensory, or behavioral difficulties (based on teachers’ reports). Most of the children came from families of middle socioeconomic background (based on parents’ occupation and education). Parental permission and ethical approval from the Research Ethics Committee of East China Normal University was obtained prior to testing.

### Materials

#### Nonverbal IQ

To assess nonverbal IQ we administered the Nonverbal Matrices task from the Das–Naglieri Cognitive Assessment System (DN CAS) battery (Naglieri and Das, 1997). This task has been used in several previous studies in Chinese showing good reliability and validity evidence (e.g., Liao et al., 2008; Deng et al., 2011). Children were presented with a page containing a pattern of shapes/geometric designs that was missing a piece and were asked to choose among five or six alternatives the piece that would accurately complete the pattern. The task was discontinued after four consecutive errors and a participant’s score was the total number correct. The Cronbach’s alpha reliability coefficient in our sample was 0.94.

#### Speed of Processing

To assess speed of processing we administered Visual Matching from the Woodcock–Johnson Tests of Cognitive Abilities (Woodcock and Johnson, 1989). Children were presented with 60 rows of numbers and were asked to cross out the two identical numbers in each row (e.g., 8, 9, 5, 2, 9, and 7) within a 3 min time limit. The first 20 rows used single-digit numbers, followed by 20 rows of two-digit numbers, and 20 rows of three-digit numbers. A participant’s score was the total number of correctly completed rows. The Cronbach’s alpha reliability coefficient in our sample was 0.84.

#### Number Sense

Number Sets was adopted from Geary et al. (2009) to assess number sense. Children were presented with four pages and each page included a target number at the top of each page (e.g., 5) and sets indicated by two or three linked boxes with Arabic numerals (e.g., 2) and concrete objects (e.g., ●●●). Children were asked to circle all the sets that can be put together to match the target number. The target number of the first two pages was 5 and the time limit was 60 s per page. The target number of the last two pages was 9 and the time limit was 90 s per page. Signal detection method was used to calculate each child’s sensitivity (*d’*) in detecting the correct sets based on the number of hits and the number of false alarms (see Geary et al., 2009, for details). The Cronbach’s alpha reliability coefficient in our sample was 0.88.

#### Executive Functioning

##### Shifting

Shifting was assessed with the Planned Connections task from the DN CAS battery (Naglieri and Das, 1997). Planned Connections is a transparent adaptation of the Trail Making task (Reitan and Wolfson, 1992). In this task, children were presented with two pages of numbers (1–14) and letters (A–N), and, in each page, they were asked to connect the numbers to the letters in successive order (1, A, 2, B, 3, C, etc.) as fast as possible. The score was the total time to finish both pages. The Cronbach’s alpha reliability coefficient in our sample was 0.80.

##### Inhibition

Inhibition was assessed with the Expressive Attention task from the DN CAS battery (Naglieri and Das, 1997). Expressive Attention is a transparent adaptation of the color-word Stroop task. Children were presented with one page of color rectangles and two pages of Chinese color characters (e.g., 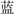
[blue], 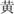
[yellow], 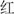
[red], 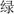
[green]). In each page, the stimuli were semi-randomly arranged in eight rows of five. Children were asked to read aloud the color of rectangles in the first page and to name the color characters in the second page as fast as possible. In the third page, children were asked to name as fast as possible the color of the ink in which the color characters were printed (e.g., the character 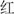
[Red] may appear in green ink) instead of saying the color character. A practice page was presented before each trial to ensure all children understood the instructions. The children’s response time on the third page was used as a measure of inhibition. The Cronbach’s alpha reliability coefficient in our sample was 0.88.

##### Working memory

The Backward Digit Span task from Wechsler Intelligence Scale for Children-Revised (Wechsler, 1974) was used to assess working memory. In this task, children were asked to repeat a sequence of digits in the reverse order. The strings of digits were presented orally by the experimenter with a time interval of about 1 s between each digit. The strings started with only two digits and one digit was added at each difficulty level (the maximum length was eight digits). The task was discontinued when participants failed both trials of a given length. A participant’s score was the maximum length of digit string recalled correctly. The Cronbach’s alpha reliability coefficient in our sample was 0.80.

#### Arithmetic Skills

##### Arithmetic accuracy

The Numerical Operations task from Wechsler Individual Achievement Test (Wechsler, 2002) was used to assess arithmetic accuracy. There were 61 problems arranged in increasing difficulty that measure arithmetic skills in basic operations (addition, subtraction, multiplication, and division) with integers and fraction, algebra, and geometry. Children were asked to write down the answer to each problem in untimed conditions. A discontinuation rule of four consecutive errors was applied and a child’s score was the total number correct. The Cronbach’s alpha reliability coefficient in our sample ranged from 0.90 to 0.94.

##### Arithmetic fluency

To assess arithmetic fluency we administered the Basic Arithmetic Test (BAT, Aunio and Räsänen, 2007, Unpublished). Children were asked to write down the answers to 28 calculation problems within a 3 min time limit. The task consisted of 28 problems: 14 additions (e.g., 2 + 1 = ? and 3 + 4 + 6 = ?) and 14 subtractions (e.g., 4 – 1 = ? and 20 – 2 – 4 = ?) that were mixed up and presented in two pages. The score was the total number correct divided by the time (in minute) to complete all items. The Cronbach’s alpha reliability coefficient in our sample ranged from 0.80 to 0.86.

### Procedure

All children were individually assessed in a quiet room at school by the first author and trained graduate students. Testing at all measurement points was completed in April/May (8–9 months after the beginning of the school year). The first testing was completed in two sessions of 30 min each. In Session A, Nonverbal Matrices, Visual Matching, Planned Connections, Expressive Attention, and Backward Digit Span were administered. In Session B, Number Sets, Numerical Operations, and BAT were administered. The order of the tasks within each session was fixed. From T2 to T4, only Numerical Operations and BAT were administered.

### Data Analysis

All measures were initially scrutinized for normality. One-way repeated-measures analysis of variance for each arithmetic skill was conducted to examine the main effects of time (linear terms) and time squared (quadratic terms). Pearson correlation coefficients were computed among all variables. Latent growth models were constructed with AMOS 17.0 to predict the growth parameters in each arithmetic skill from the six predictor variables measured. Full information maximum likelihood method was applied to make full use of the data.

## Results

### Preliminary Data Analysis

Descriptive statistics for all the measures used in our study are shown in **Table 1**. An examination of the distributional properties of the measures revealed that they were within acceptable levels (Tabachnick and Fidell, 2007). The results of one-way repeated-measures analysis of variance for each mathematics skill showed a significant main effect of linear terms of time (for arithmetic accuracy, *F*(1,163) = 951.19, *p* < 0.001; for arithmetic fluency, *F*(1,162) = 538.85, *p* < 0.001), and a non-significant main effect of quadratic terms of time (for arithmetic accuracy, *F*(1,163) = 1.91, *p* > 0.05; for arithmetic fluency, *F*(1,163) = 2.16, *p* > 0.05), which indicated a linear growth trend for both mathematics skills.

**Table 1:** Descriptive statistics for all variables used in the present study.

	*M*	*SD*	*Min.*	*Max.*
Nonverbal IQ	19.77	4.15	12	32
Speed of processing	38.47	5.15	24	51
Number sense	3.14	0.46	1.80	3.83
Inhibition	68	17.25	31	130
Shifting	136.50	46.67	52	292
Working memory	4.13	1.26	2	8
Arithmetic accuracy T1	23.08	2.18	18	31
Arithmetic accuracy T2	28.84	3.14	21	36
Arithmetic accuracy T3	33.49	4.91	25	48
Arithmetic accuracy T4	38.70	7.02	26	54
Arithmetic fluency T1	12.96	3.04	6.96	22.82
Arithmetic fluency T2	15.44	3.90	7.73	31.26
Arithmetic fluency T3	17.88	4.45	8.29	32.36
Arithmetic fluency T4	19.89	4.65	8.67	35.25

### Correlations Among All the Measures

**Table 2** shows the results of the correlational analysis. There was moderate to high stability between all measurement points for arithmetic accuracy (the correlations ranged from 0.35 to 0.61), and high stability between all measurement points for arithmetic fluency (the correlations ranged from 0.54 to 0.67). Besides, arithmetic accuracy correlated significantly with arithmetic fluency at all measurement points. Nonverbal IQ, speed of processing, number sense, inhibition, and working memory at T1 correlated significantly with arithmetic accuracy at all measurement points (absolute *r*s values ranged from 0.15 to 0.36), and shifting correlated significantly with arithmetic accuracy at T3 and T4. Speed of processing, number sense, and inhibition at T1 correlated moderately with arithmetic fluency at all measurement points (absolute *r*s values ranged from 0.30 to 0.43). Finally, working memory at T1 correlated weakly with arithmetic fluency at T2 and T4, and shifting correlated weakly with arithmetic fluency at T3.

**Table 2:** Correlations between cognitive predictors and mathematics outcomes.

	1	2	3	4	5	6	7	8	9	10	11	12	13
(1) Nonverbal IQ													
(2) Speed of processing	0.22												
(3) Number sense	0.26	0.39											
(4) Inhibition	–0.25	–0.43	–0.32										
(5) Shifting	–0.21	–0.38	–0.42	0.30									
(6) Working memory	0.23	0.26	0.16	–0.29	–0.07								
(7) Arithmetic accuracy T1	0.23	0.28	0.21	–0.19	–0.09	0.20							
(8) Arithmetic accuracy T2	0.33	0.28	0.26	–0.15	–0.14	0.25	0.44						
(9) Arithmetic accuracy T3	0.36	0.22	0.25	–0.20	–0.17	0.22	0.43	0.55					
(10) Arithmetic accuracy T4	0.33	0.20	0.28	–0.17	–0.21	0.34	0.35	0.51	0.61				
(11) Arithmetic fluency T1	0.06	0.42	0.37	–0.33	–0.14	0.15	0.30	0.38	0.23	0.31			
(12) Arithmetic fluency T2	0.17	0.40	0.30	–0.33	–0.07	0.22	0.34	0.34	0.35	0.29	0.57		
(13) Arithmetic fluency T3	0.19	0.43	0.35	–0.34	–0.16	0.14	0.44	0.31	0.32	0.31	0.60	0.62	
(14) Arithmetic fluency T4	0.18	0.38	0.34	–0.37	–0.13	0.31	0.36	0.36	0.32	0.30	0.54	0.60	0.67

*Correlations lower than 0.16 were not significant. Correlations between 0.16 and 0.20 were significant at the 0.05 level and correlations higher than 0.20 were significant at the 0.01 level.*

### Latent Growth Models for Arithmetic Skills

First, unconditional latent linear growth models (without any predictors) were constructed, in which the intercept represents the arithmetic skill at T1, and the slope represents the rate of linear growth from T1 to T4. The model for arithmetic fluency showed a good fit, χ^2^ = 4.55, *df* = 5, *p* = 0.47, CFI = 1.000, TLI = 1.003, RMSEA = 0.000, and the correlation between the intercept and slope was not significant (estimated *r* = 0.31, *p* > 0.12). In turn, the model for arithmetic accuracy did not fit the data well. The modification indices indicated that the estimated residual of arithmetic accuracy at T3 was related to that of T4, suggesting that the two measurements shared some unique variance that was not included in the model. After incorporating the above relation in the model, the model fit the data very well, χ^2^ = 7.43, *df* = 4, *p* = 0.12, CFI = 0.981, TLI = 0.953, RMSEA = 0.069, and the correlation between the intercept and slope was significant (estimated *r* = 0.82, *p* < 0.05). The results also showed a significant variance in the intercepts and slopes of both mathematics skills (for arithmetic accuracy, σ_i_^2^ = 1.74, *p* < 0.05, σ_s_^2^ = 1.36, *p* < 0.05; for arithmetic fluency, σ_i_^2^ = 6.29, *p* < 0.001, σ_s_^2^ = 0.66, *p* < 0.01).

**FIGURE 1:**
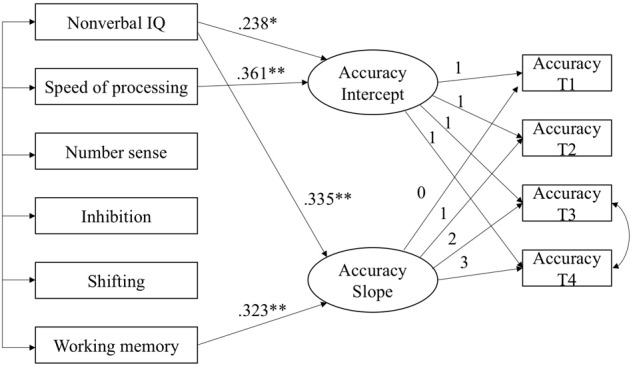
Predicting the intercept and slope in arithmetic accuracy. Model Fit: χ^2^ = 14.97, df = 16, *p* = 0.53, CPI = 0.935, TLI = 1.010, RMSEA = 0.000, ^∗^p < 0.05, ^∗∗^p < 0.01.

**FIGURE 2:**
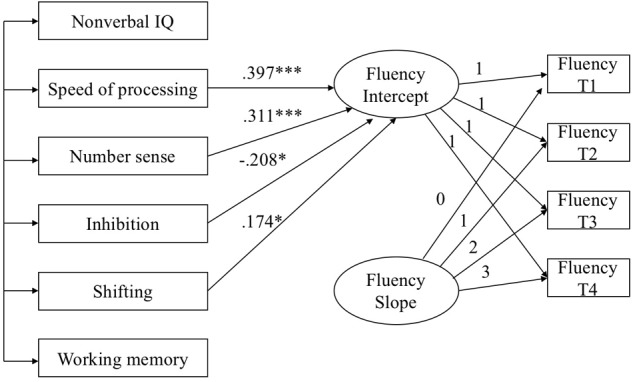
Predicting the intercept and slope in arithmetic fluency. Model Fit: χ^2^ = 16.63, df = 17, *p* = 0.48, CFI = 1.000, TLI = 1.002, RMSEA = 0.000, ^∗^p < 0.05, ^∗∗∗^p < 0.01.

Next, six variables at T1 were used to predict the intercept and slope of a linear growth model for each mathematics skill. In both models, the intercept was allowed to correlate with the slope, and the residuals of the predictors were allowed to be correlated. The models predicting growth in arithmetic accuracy and arithmetic fluency are shown in **Figures 1, 2**, respectively, with non-significant paths removed. Both models fit the data well (for arithmetic accuracy, χ^2^= 14.97, *df* = 16, *p* = 0.53, CFI = 0.935, TLI = 1.010, RMSEA = 0.000; for arithmetic fluency, χ^2^= 16.63, *df* = 17, *p* = 0.48, CFI = 1.000, TLI = 1.002, RMSEA = 0.000). Nonverbal IQ and speed of processing predicted the intercept of arithmetic accuracy and accounted for 36.4% of the variance. Nonverbal IQ and working memory predicted the slope of arithmetic accuracy and accounted for 31.3% of the variance. Speed of processing, number sense, inhibition and shifting predicted the intercept of arithmetic fluency and accounted for 39.6% of the variance. No variables predicted significantly the slope of arithmetic fluency.

## Discussion

This study aimed to examine how the three core EF subcomponents (i.e., inhibition, shifting, and working memory) predict the growth parameters of two mathematics skills (i.e., arithmetic accuracy and fluency) in a group of Chinese children followed from Grade 2 to 5. The results showed that the three EF subcomponents were interrelated, but predicted different growth parameters in different mathematics skills. Whereas working memory uniquely predicted the slope of arithmetic accuracy, inhibition and shifting predicted the intercept of arithmetic fluency.

In contrast to our expectation (see Hypothesis 1) and to the findings of some previous studies (e.g., Viterbori et al., 2015; Cragg et al., 2017), working memory did not uniquely predict the growth parameters of arithmetic fluency. This may be due to the fact that neither Cragg et al. (2017) nor Viterbori et al. (2015) controlled for number sense and/or speed of processing before examining the contribution of working memory to arithmetic fluency. However, it may also reflect differences in the amount of working memory involved in the strategies used to solve simple calculations in different countries. Children in North America learn how to solve simple calculations in Grade 1 and by Grade 2 (when we first assessed them) they still use “immature” strategies (e.g., counting on) that tax working memory (e.g., Geary et al., 1996; Bailey et al., 2012; see also Miller et al., 2005, for a review of differences in how children learn mathematics in China and the United States). In contrast, in China, Grade 2 children solve simple calculations by retrieving the answer from their long-term memory. This is because they have been practicing simple calculations since the age of 3 (when they go to kindergarten).

Second, our results showed that working memory uniquely predicted the slope in arithmetic accuracy (see Geary, 2011; Viterbori et al., 2015, for a similar finding). This suggests that working memory contributes to the learning of new operations, which are basic operations in lower grades and more complex operations in higher grades. Geary (2011) and Yen et al. (2017) also argued that EF may be more important in higher grades, because more complex and difficult operations need the extensive engagement of central executive. Once the operation and calculation reaches an automatic level, working memory may no longer have a role to play in the calculation process (Träff, 2013; Cowan and Powell, 2014).

In line with our second hypothesis, we also found that inhibition uniquely predicted the intercept in arithmetic fluency even after controlling for the effects of nonverbal IQ, speed of processing, and number sense. Viterbori et al. (2015) have argued that inhibition may be involved in the process of retrieving the arithmetic facts and is required for suppressing competing responses. For example, when retrieving the answer 5 in response to 3 + 2, children need to suppress 6 as the solution to 3 × 2, considering that single digit multiplication is learned by most of Chinese children through rote memory (Zhou et al., 2006).

In contrast to our third hypothesis as well as to the findings of previous studies (e.g., Cantin et al., 2016; Cragg et al., 2017; Simanowski and Krajewski, 2017), shifting was a significant predictor of the intercept in arithmetic fluency. A possible explanation may be that we used time scores of shifting, while Cantin et al. (2016) and Cragg et al. (2017) used accuracy scores. It may also be due to the task we used to operationalize arithmetic fluency. Specifically, because BAT mixes up the addition and subtraction problems, children likely had to switch between addition and subtraction mindsets.

However, neither inhibition nor shifting predicted the slope of arithmetic fluency. Because Chinese children learn different calculations when they go to kindergarten (at the age of 3), by the time they reach elementary school they have already mastered simple calculations. Subsequently, when asked to perform simple calculations they rely more on fact retrieval than on procedural strategies (e.g., Geary et al., 1996; Bailey et al., 2012; Vanbinst et al., 2015). Inhibition and shifting may be important in arithmetic fluency in China but in earlier grades when Chinese children learn to perform simple calculations (i.e., the 3 years of kindergarten).

Some limitations of the present study are worth mentioning. First, we used single measures of each EF subcomponent and this may have weakened each construct and subsequently its contribution to mathematics. Future studies should assess each EF component with more tasks. Second, in order to directly compare the contribution of EF subcomponents to timed and untimed mathematics skills, we did not include problem solving since problem solving is a higher-level mathematics skill predicted not only by domain-general skills, but also by reading-related skills (e.g., Andersson, 2008; Träff, 2013). Third, our measures of working memory and shifting involved numerical stimuli. This may have increased the contribution of their respective constructs to mathematics. However, notice that we controlled for the effects of other cognitive skills that also contained numerical stimuli (e.g., speed of processing, number sense). Fourth, we did not obtain information on family’s income. Some studies (e.g., Hackman et al., 2015; Chung et al., 2017) have shown that family’s income correlates with both EF and children’s math achievement. This implies that the relationship between EF and mathematics might be due to family’s income. Future studies should explore this possibility. Fifth, due to time restrictions, we took a purely cognitive view of mathematics. We acknowledge that affective, social, and emotional attributes may play an equally strong role in mathematics development. Finally, although many Chinese parents pay private tutors (typically from commercial education companies) to instruct their children to practice mathematical skills with more homework, we were not able to obtain information on this issue and, as a result, we were not able to control for its effects on mathematics skills.

## Conclusion

Our study adds to a growing body of research on the contribution of different EF subcomponents to mathematics development (e.g., van der Ven et al., 2012; Van de Weijer-Bergsma et al., 2015; Lee and Bull, 2016) suggesting that different EF subcomponents may contribute to different growth parameters in arithmetic accuracy and fluency, even after controlling for the effects of other known predictors of mathematics (i.e., nonverbal IQ, speed of processing, and number sense). We echo here Cragg and Gilmore’s (2014) conclusion that different EF skills contribute to different components of mathematical knowledge as well as Miyake et al.’s (2000) conclusion that the unity of the EF subcomponents is important but it is diversity in what skills they predict that makes things interesting. From a practical point of view, this suggests that depending on what mathematics outcome we want to predict we should include different types of EF tasks to maximize our predictive power. At the same time, this finding implies that depending on the type of mathematics difficulties a child has (e.g., procedural vs. semantic memory difficulties; Geary, 1993) and to the extent we want to provide an EF intervention (see Dias and Seabra, 2017), we need to focus on different EF subcomponents to maximize our chances to be effective.

## Author Contributions

CD, GG, WW, and AT designed the study. WW and LG collected the data, prepared the data for analysis, and wrote the manuscript. All authors interpreted the data and discussed the results. GG, CD, WW, and LG revised the manuscript.

## Conflict of Interest Statement

The authors declare that the research was conducted in the absence of any commercial or financial relationships that could be construed as a potential conflict of interest.
